# Morbidity and Mortality of Cytoreductive Surgery with Hyperthermic Intraperitoneal Chemotherapy: National Cancer Institute, Mexico City, Mexico

**DOI:** 10.5402/2011/526384

**Published:** 2011-08-03

**Authors:** Horacio N. López-Basave, Flavia Morales-Vásquez, J. M. Ruiz Molina, Aaron González-Enciso, Silvio A. Ñamendys-Silva, Juan M. Medina Castro, Gonzalo Montalvo-Esquivel, Angel Herrera-Gómez, Jaime G. De la Garza Salazar

**Affiliations:** ^1^Department of Surgical Oncology, National Cancer Institute, San Fernando No. 22 Colonia Seccion XVI, Tlalpan, 14080 Mexico City, DF, Mexico; ^2^Department of Medical Oncology, National Cancer Institute, San Fernando No. 22 Colonia Seccion XVI, Tlalpan, 14080 Mexico City, DF, Mexico; ^3^Department of Critical Care Medicine, National Cancer Institute, San Fernando No. 22 Colonia Seccion XVI, Tlalpan, 14080 Mexico City, DF, Mexico

## Abstract

Peritoneal carcinomatosis (PC) is generally considered a lethal disease, with a poor prognosis. Cytoreductive surgery (CRS) with hyperthermic intraperitoneal chemotherapy (HIPEC) has emerged as a new approach for peritoneal surface disease. This study investigated the early experience with this combined modality treatment at a single institute. From January 2007 to March 2010, 24 patients were treated After aggressive CS, with HIPEC (cisplatin 25 mg/m^2^/L and mitomycin C 3.3 mg/m^2^/L was administered for 90-minutes at 40.5° C). These data suggest that aggressive CRS with HIPEC for the treatment of PC may result in low mortality and acceptable morbidity. Rigorous patient selection, appropriate and prudent operative procedures were associated with encouraging results in our experience.

## 1. Introduction

The term peritoneal carcinomatosis (PC) was first used by Sampson in 1931 to describe a common manifestation of tumor progression in gastrointestinal and gynecologic malignancies [1]. PC is characterized by the presence of numerous tumour nodules of various sizes that are distributed throughout the abdominal-pelvic cavity. As the disease progresses, the nodules become confluent to form plaques, masses, or uniformly cover peritoneal surfaces. The same clinical picture may be seen in rare primary peritoneal tumors, such as peritoneal mesothelioma and serous-papillary peritoneal (extraovarian) carcinoma [2]. 

Until recently, PC has been considered a systemic metastatic condition with extremely poor prognosis and no standard therapy. These patients are generally managed by supportive or palliative care and systemic chemotherapy. The role of surgery has been traditionally limited to the palliation of bowel obstruction or painful tumor masses, with unsatisfactory results in terms of survival benefit, quality of life, or disease control. In large historical series, a median survival of 5–7 months has been reported for patients with PC of colorectal origin and about 3 months for patients with PC of gastric origin [3, 4]. 

In the last two decades, better understanding of the natural history and biology of PC has occurred. The evidence that the disease remains frequently confined to the peritoneal surface with no hepatic or distant metastases has evolved into an aggressive clinical approach focused on providing the greatest overall benefit for these patients. Cytoreductive surgery (CRS) with hyperthermic intraperitoneal chemotherapy (HIPEC) is a multimodality treatment aiming at complete disease eradication through the surgical cytoreduction of all the visible tumor in combination with HIPEC, to eradicate the microscopic residual disease [5, 6].

Although pseudomyxoma peritonei is a locally aggressive not metastasizing condition originating from low-grade appendiceal neoplasms, long-term survival of only 20–30% has been reported after debulking surgery and palliative chemotherapy. With the advent of the local-regional approach, five-year and median survival have increased to 52–96% and 51–156 months, respectively [7]. This has stimulated the use of CRS and HIPEC in other peritoneal surface malignancies. Several centers have reported a median survival improvement up to 34–92 months for peritoneal mesothelioma and up to 38–62 months for colorectal cancer PC [8–11]. In stage-III ovarian cancer, complete surgical cytoreduction has been shown to be closely related to survival and a phase-III study has demonstrated the survival benefit of intraperitoneal versus intravenous chemotherapy, resulting in a growing interest for the use of the combined treatment in this clinical setting [12–14].

Due to treatment complexity, CRS with HIPEC is associated with high operative morbidity and significant economic costs. As a consequence, the slow diffusion of this technique in many countries precludes most patients from being referred for appropriate treatment [15, 16]. The National Cancer Institute (INCAN) in Mexico City is a comprehensive cancer centre and the national reference institution for oncology research and clinical practice. We started our peritoneal surface malignancy program in January 2007. This current study was carried out to critically analyze the morbidity and mortality of our initial experience with cytoreductive surgery and HIPEC. Additionally, patient survival was assessed as secondary end-point. These early results are presented in an attempt to document the efforts required to initiate, develop, and maintain a peritoneal surface malignancy management unit, with the hope that such an experience may be of help to others. 

## 2. Material and Methods

All patients were treated following a feasibility protocol approved by the Institutional Scientific Committee and Bioethics Committee. An informed consent form was signed by all patients. The trial started in January 2007.

### 2.1. Patient Selection

Eligibility criteria for combined treatment the were the following:

1. clinical, radiological, or pathological diagnosis of peritoneal carcinomatosis,
2. age 18 to 75 years,
3. no concomitant severe heart, respiratory, liver, hematologic, or kidney diseases,
4. functional status of 0–2 according to the Eastern Cooperative Oncology Group (ECOG),
5. absence of hepatic or extra-abdominal metastatic disease,
6. PC amenable to potentially complete surgical cytoreduction at preoperative computed tomography scan.

Evaluation for suitability to undergo CRS and HIPEC was made during multidisciplinary meetings attended by surgical oncologists, medical oncologists, radiologists, cancer care nurses, and research staff.

### 2.2. Cytoreductive Surgery

The goal of the surgical cytoreduction was to remove all the visible disease, according to the technique originally described by Sugarbaker [6]. Briefly, with the patient in a supine position, a xiphoid-pubic midline incision was made. The cavity was reviewed to determine the extent of disease and feasibility of a complete cytoreduction. The “peritoneal cancer index” (PCI), which integrates size and distribution in the peritoneal surface, was used to determine the extent of the tumour [17]. In all patients, the diagnosis of PC was confirmed by frozen section pathological examination; peritoneal lavage for cytology was performed using 400 mL sterile solution (100 mL in each quadrant). 

The cytoreductive surgical procedures included one or more of the following steps, depending on the extent of disease:

1. greater omentectomy, right parietal peritonectomy and right colon resection,
2. pelvic peritonectomy, resection of the sigmoid colon with total abdominal hysterectomy and salpingo-oophorectomy in women,
3. omentectomy, extensive dissection of the hepatic ligament, cholecystectomy with or without gastric antrectomy,
4. right upper quadrant peritonectomy and Glisson's capsule resection,
5. left upper quadrant peritonectomy and splenectomy,
6. other intestinal resection and/or abdominal mass resection.

Completeness of cytoreduction (CCR) was classified at the end of the surgical phase according to Sugarbaker criteria, as macroscopically complete (CCR-0); optimal: residual disease ≤2.5 mm in any region (CCR-1); or grossly incomplete: residual disease >2.5 mm (CCR-2) or >25 mm (CCR-3) [17].

### 2.3. HIPEC Technique

HIPEC was performed at the end of the surgical cytoreduction and after the completion of bowel anastomoses, with verification of haemostasis. Only patients who were able to be completely or optimally cytoreduced, with no retroperitoneal nodal involvement, with hemodynamically stable conditions at the end of the surgery and did not suffer of major intraoperative complications, underwent HIPEC. Since a possible adverse effect of heat and local-regional chemotherapy has been reported, HIPEC was performed only in patients receiving not more than 2 bowel anastomoses.

Four in-flow catheters were placed in each abdominal quadrant. A fifth out-flow catheter was placed in the central abdomen to collect the perfusate. Chemotherapy agents used were mitomycin-C (3.3 mg/m^2^/L) and cisplatin (25 mg/m^2^/L) for 90 minutes [18]. The HIPEC was conducted according to the closed-abdomen technique in 23 patients, with temporary closure of the skin and reopening of the abdomen at the end of the perfusion for checking the whole cavity before definitive closure. In one patient, the procedure was performed according to the Coliseum technique, by elevating the skin edges and covering the open abdominal cavity with a plastic sheet. An extracorporeal circulation device with a heat exchanger was used. Perfusate volume was 3-4 L. The perfusion solution was kept at an average of 41°C (range 40–43°C). Intracavitary, out-flow and in-flow catheter temperatures were continuously monitored. Once the temperature was uniform in all quadrants, the total dose of the chemotherapy was fractionated in 3 portions: 50%, 25%, and 25%.; this dose was perfused 30 minutes each one.

### 2.4. Statistics

Continuous variables were expressed as means and range, and categorical variables as frequency and percentages. In operative morbidity analysis, both surgical complications and systemic toxicity related to local-regional chemotherapy were considered. Postoperative complications occurring within 30 days of the procedure were scored according to the National Cancer Institute Common Terminology Criteria (http://ctep.cancer.gov/forms/CTCAEv3.pdf). The following independent variables were taken into consideration for potential association with major surgical complications: primary tumor histology, gender, functional status, age, body mass index, prior chemotherapy, prior radiotherapy, duration of procedure, and the extent of cytoreduction. Survival curves were estimated using the Kaplan-Meier method [19]. The log-rank test was used to compare overall survival data. Statistical significance was defined as *P* < 0.05. Statistical analyses were performed using the Statistical Package for the Social Sciences software (version 15.0; SPSS, Chicago, IL, USA).

## 3. Results

From January 2007 to March 2010, a total of twenty-four combined procedures were performed in 20 women and four men. Primary cancer was epithelial ovarian cancer in ten cases, colorectal cancer in six, appendiceal carcinoma in three, gastric cancer in two, and three patients had pseudomyxoma peritonei originating from low-grade mucinous tumor. Patient characteristics are summarized in Table 1. 

### 3.1. Operative Outcomes

Average operative time was 402 minutes (range of 350–640 minutes). The average blood loss volume was 938 mL, ranging between 100 and 3700 mL with an average transfusion of 3 packages of red blood cells per patient (range 1–5). 

The following intraoperative complications occurred and were managed during the operation: diaphragm opening (*n* = 4) and intestinal perforation (*n* = 1) requiring immediate repair. 

Thirteen of the 24 patients (54%) included in the study required postoperative admission to the intensive care unit (ICU). The average length of stay in ICU was 1.7 days (range of 1–13 days). The average length of stay in hospital was 8.2 days (range of 5–19 days).

Major postoperative complications (Table 2) (grades 3-4) occurred following 5 combined procedures (20.8%), including bleeding (*n* = 2), pneumonia (*n* = 1), fistula (*n* = 1), and acute renal failure (*n* = 1). Return to the operation theater for control of postoperative bleeding was needed in two patients, accounting for a reoperation rate of 8.33%. No operative death occurred.

### 3.2. Survival and Failures

**Figure 1:**
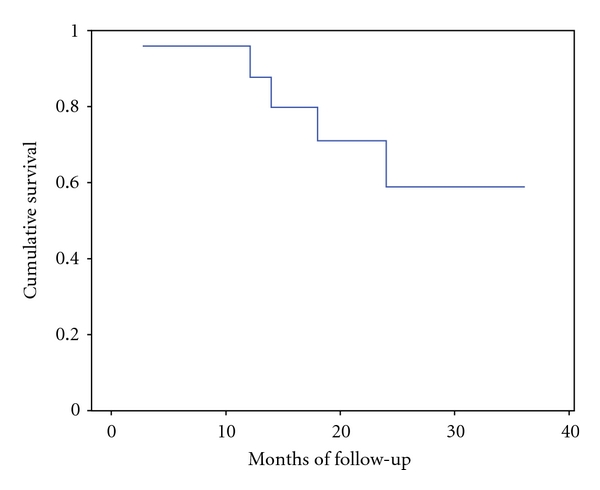
Overall survival of the entire study group.

For the overall series, median followup was 15.5 months (range 3–36). The Kaplan-Maier estimated survival of the entire series is shown in Figure 1. At the end of the study period, 22 patients were alive and 2 died for disease-related causes, accounting for a median survival of 28 months. 

**Figure 2:**
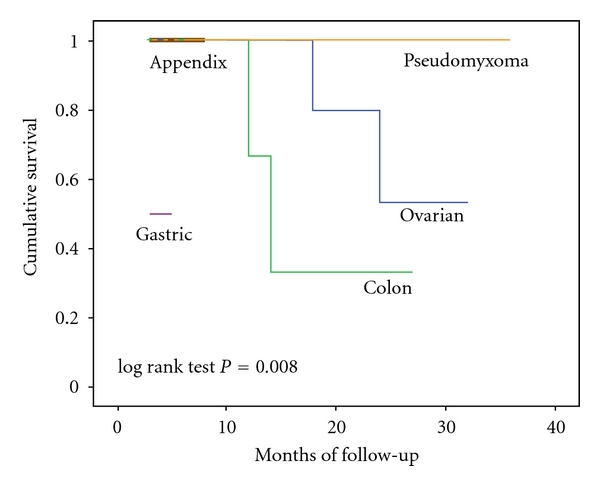
Overall survival according to primary tumor.

In a subgroup analysis, all the patients with pseudomyxoma peritonei (*n* = 3), gastric cancer (*n* = 2), and appendiceal carcinoma (*n* = 3) were alive at a median of 25, 11, and 8 months, respectively. Among patients treated for PC of colorectal cancer, four are currently alive at a median of 11 months and two died at 11 and 12 months from CRS and HIPEC. Finally, as patients with epithelial ovarian cancer are concerned, eight are currently alive at a median of 20 months and two died at 12 and 24 months from combined treatment. Overall survival curves according to primary tumor are shown in Figure 2.

## 4. Discussion

Cancer is a severe public health problem, and peritoneal carcinomatosis is one of the most common cause of death in patients affected by intra-abdominal cancers [6]. PC is diagnosed in 8–10% of cases at the time of the initial diagnosis of primary colorectal tumor, and in 13–30% of patients with recurrent disease; in 25–35% of cases, PC is confined exclusively to the peritoneum [20]. Gastric cancer is still an important gastrointestinal malignancy worldwide, especially in developing country. Peritoneum is the first site of treatment failure in up to 40–50% of patients [10]. Epithelial ovarian cancer is diagnosed at an advanced stage in up to 75% of women, with peritoneal involvement and/or distant or nodal metastases (International Federation of Gynecology and Obstetrics stage III/IV) [12]. 

Conventional treatment options for PC, such as supportive care, palliative or debulking surgery, and systemic chemotherapy, do not provide adequate benefit to these patients [3, 4]. With this in mind, researchers have been working during the last decades on an innovative combined treatment modality. In 1982, Pestieau and Sugarbaker proposed the management of PC by peritonectomy procedures and multivisceral resections to maximally cytoreduce the macroscopic disease, in combination with intraperitoneal hyperthermic chemotherapy to treat the residual microscopic disease [21]. CS creates the optimal environment for intraperitoneal chemotherapy, and local-regional drug administration results in higher intraperitoneal concentrations and minimal systemic toxicity. The intraoperative time setting allows optimal distribution throughout the abdominal cavity before the development of postoperative adhesions. Finally, mild hyperthermia has both intrinsic and synergistic effects with platinum compounds and mitomycin-C. Taken together, these concepts represent the rational bases of comprehensive treatment [5, 6].

Several independent trials of CRS and HIPEC have reported a dramatic survival improvement in selected patients with peritoneal surface malignancies [7–11, 14]. Furthermore, three comparative studies have demonstrated the superiority of the combined approach over conventional therapies in the treatment of colorectal cancer PC. In a randomized trial, median survival was 23 months with CS and HIPEC and 12.6 months with fluorouracil/leucovorin-based systemic chemotherapy (*P* = 0.0032) [22]. In a French retrospective controlled study, median survival was 23.9 months with modern systemic agents and an unprecedented 62.7 months with optimal cytoreduction and HIPEC (*P* < 0.05) [23]. Finally, Franko et al. has recently reported a median survival of 34.7 months in 67 patients treated with CRS and HIPEC, as compared to 16.8 months in a matched control group managed by modern systemic chemotherapy ± biological agents (*P* < 0.001) [24].

CRS and HIPEC is a complex procedure involving extensive stripping of the peritoneal surface, multiple visceral resection, up to 4-5 bowel anastomoses, and high-dose chemotherapy under hyperthermic conditions. Operative time of 10–14 hours is the rule [7–11]. High rates of potentially life-threatening complications have been reported by all the centres managing these patients [25]. Reported morbidity and mortality rates vary widely from 12% to 67.6% and 0% to 9%, respectively. The most common adverse events are intestinal perforations, anastomotic dehiscence, intestinal fistula, bile leak, postoperative bleeding, and pancreatitis; in addition to the usual major surgical risks: deep vein thrombosis, pulmonary embolism, pneumothorax, myocardial infarction, bone marrow aplasia, and haematological toxicity. The complications may be secondary to surgery, hyperthermia, and chemotherapy. 

This paper describes the initial experience with peritonectomy and intraoperative intraperitoneal chemotherapy infusion in a Mexican tertiary referral center. In the current series, morbidity rate was 20.8% with 5 patients who developed complications. No operative death occurred. The adverse event rate in the current study is in line with that reported by the international centers performing the combined procedure [25]. Recent reports suggest that the initial high morbidity associated to CRS and HIPEC decreases with increasing experience. This is most marked in specialized centers and includes improvements in patient selection, surgical expertise, and postoperative management. This phenomenon has been termed “learning curve” and reflects the difficulties in reaching a high level of performance in demanding surgical procedures [25–27]. 

Moran analyzed the factors that influenced the learning curve for CRS and HIPEC primarily for PMP [26, 27]. The first 100 patients treated were divided into 3 numerically equal groups of 33, 33, and 34, respectively. Thus, his 6-year experience was divided into 3-time intervals of 79, 16, and 9 months. Both major morbidity and mortality fell with increasing experience, with anastomotic leakage and reoperation for bleeding occurring predominantly during the initial period. Anastomotic leak rate fell from 12% to 0%, and reoperation for bleeding from 15% to 0% over the whole 6-year period.

In our experience, patient selection through periodic multidisciplinary meetings has been an important element in reducing complication rates during the initial phase. The involvement of experienced surgeons, as well as other health care specialists, has been effective to facilitate training, experience, and the management of adverse events. Furthermore, knowledge of the toxicity of HIPEC, in particular the increased risks of anastomotic leakage likely related to hyperthermic chemotherapy, has resulted in a low threshold for dose modification and even withdrawal from HIPEC, in patients at high risk of perioperative morbidity or mortality. Finally, rigorous data collection has been essential to facilitate service improvement and improve the safety of a high-risk intervention such as CRS with HIPEC. 

There have been varying morbidity and mortality rates in published data [2]. This may be in part explained by the difference among groups in terms of eligibility criteria, surgical aggressiveness, technical skills, modality of delivering the local-regional chemotherapy and the lack of a standardized adverse event classification and scoring system. In 1999, Stephens et al. carried out a study involving 200 patients and reported 27% morbidity with 6% peripancreatitis, and a mortality of 1.5% [28]. In 2006, Smeenk et al. [29] reported 54% toxicity and 3% mortality in 103 procedures of pseudomyxoma peritonei. A univariate analysis found statistical significance between toxicity and age, perforation of the small intestine, and abdominal tumor burden. In the current study, in agreement with previously published data, greater morbidity was found in patients with bulkier disease who required longer operating time and in whom there was more bleeding [9, 11, 23–25].

In conclusion, our data demonstrated that an appropriately rigorous approach to CRS and HIPEC may result in the initiation and development of a new service with low mortality, acceptable morbidity and encouraging survival results. Great attention to patient selection, team work awareness of technical complexity, knowledge of the most common morbidity patterns, and willingness to learn from established units have been effective in improving the learning curve.

## Figures and Tables

**Table 1:** Characteristics of the study patients.

Age, average (range)	55.4 (26–68)
Gender	
Female,	20 (87.5%)
Male	4 (12.5%)
Primary site	
Ovarian epithelia cancer	10
Colorectal	6
Appendix	3
Pseudomyxoma	3
Gastric	2
Previous surgery	24 (100%)
Previous systemic chemotherapy	21 (87.5%)
Tumours	10 yes (42%): CEA
marker	14 No (58%)

**Table 2:** Complications.

Complications	9 cases (37%)	2 bleeding
		1 pneumonia 1 Fístula
		1 acute renal failure
		4 diaphragm opening
	15 no (%)	

Reoperation	2 cases (8.33%)	Bleeding into the operated site


Intensive care	13 yes (54%)	1 bleeding
		5 extended surgery time
	11 no (46%)	4 diaphragm opening

Mortality	0	No
